# A tumor endothelial cell-specific microRNA replacement therapy for hepatocellular carcinoma

**DOI:** 10.1016/j.isci.2024.108797

**Published:** 2024-01-04

**Authors:** Hideki Iwamoto, Hiroyuki Suzuki, Atsutaka Masuda, Takahiko Sakaue, Toru Nakamura, Toshimitsu Tanaka, Miwa Sakai, Yasuko Imamura, Hirohisa Yano, Takuji Torimura, Hironori Koga, Kaori Yasuda, Masakatsu Tsurusaki, Takahiro Seki, Takumi Kawaguchi

**Affiliations:** 1 Division of Gastroenterology, Department of Medicine, Kurume University School of Medicine, Kurume 831 0011, Japan; 2 Liver Cancer Research Division, Research Center for Innovative Cancer Therapy, Kurume University School of Medicine, Kurume 831 0011, Japan; 3 Department of Medicine, Iwamoto Internal Medicine Clinic, Kitakyushu 802 0832, Japan; 4 Department of Pathology, Kurume University School of Medicine, Kurume 830-0011, Japan; 5 Cell Innovator, Inc., Venture Business Laboratory of Kyushu University, Fukuoka 812-8582, Japan; 6 Department of Radiology, Kindai University Faculty of Medicine, Osaka-Sayama 589 8511, Japan; 7 Department of Microbiology, Tumor and Cell Biology, Karolinska Institutet, 17165 Solna, Sweden

**Keywords:** Molecular biology, Cancer

## Abstract

Current approved anti-angiogenic drugs (AAD) for hepatocellular carcinoma (HCC) inhibit tumor angiogenesis, but affect the hepatic vasculature resulting in adverse effects. Tumor endothelial cells (TECs) differ from normal endothelial cells. In this study, we aimed to detect TEC-specific miRNAs and develop an anti-angiogenic treatment specific for TECs. We established HCC orthotopic mouse models. TEC-specific miRNAs were detected using a microRNA array. Finally, we evaluated the therapeutic effects of the TEC-specific miRNA agonist cocktail. In total, 260 TEC-specific genes were detected. Among the top ten downregulated TEC-specific miRNAs, miR-139-3p and 214-3p were important for the TEC phenotype. The TEC-specific microRNA agonist cocktail showed significant anti-tumor effects by inhibiting tumor angiogenesis without affecting hepatic vasculatures in HCC orthotopic mouse models. Moreover, it significantly downregulated tip-cell sprouting-related genes. We identified two downregulated TEC-specific miRNAs; microRNA replacement therapy, which targets the downregulated TEC-specific miRNAs, is an effective and promising treatment for HCC.

## Highlights

• miR-139-3p and miR-214-3p were important for the phenotype of tumor vessels
• TEC-specific miRNA replacement therapy only inhibited tumor angiogenesis
• TEC-specific miRNA replacement therapy downregulated genes regarding vascular sprouting

## Graphical abstract

**Figure:**
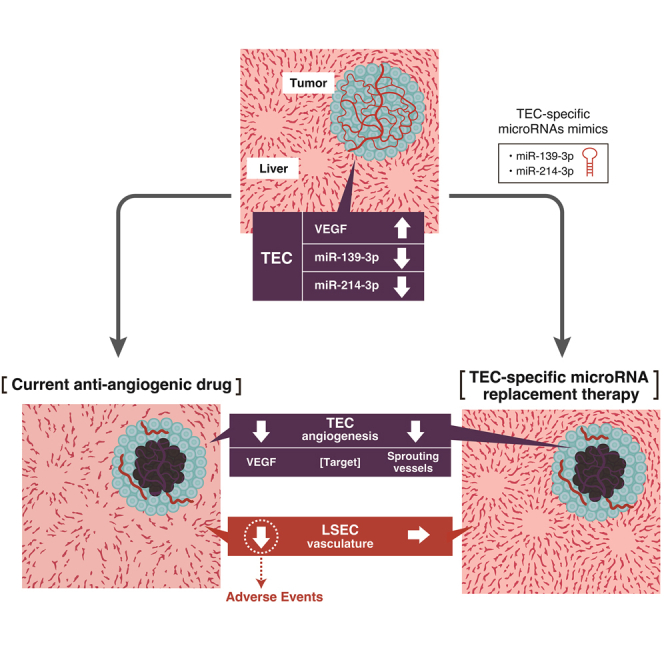

## Introduction

Systemic treatments for hepatocellular carcinoma (HCC) have developed remarkably.^1^ Drugs used in HCC treatment mainly target cells composed of the tumor microenvironment, such as tumor endothelial cells (TECs) and immune-related cells.^2^ Molecular targeted therapy targeting tumor angiogenesis has become the main systemic treatment for various cancer types, including HCC.^3^ Current anti-angiogenic drugs (AADs) inhibit angiogenesis by inhibiting the vascular endothelial growth factor (VEGF) signaling pathway.^2^ Although anti-angiogenic therapy has contributed to improving the survival of patients with cancer, many clinical unmet needs, such as the appearance of adverse events and acquired resistance, still exist. Current AADs have a high incidence of adverse events because of their non-specificity for TECs. Currently, AADs commonly inhibit VEGF signaling. VEGF is essential for tumor angiogenesis and maintaining vascular structures in healthy organs.^4^ We have previously reported that an anti-VEGF antibody or sunitinib, a tyrosine kinase inhibitor that targets the VEGF receptor, inhibited tumor angiogenesis and vascular structures of endocrine organs such as the thyroid, which was correlated with the appearance of hypothyroidism.^5^ VEGF blockade also affects the hepatic vasculature. We found that VEGF blockade induced a reduction in hepatic vasculatures and morphological changes in liver sinusoidal endothelial cells (LSEC), which resulted in the appearance of adverse events and the acceleration of metastasis in cancer cells.^6^ Although current AADs are effective drugs, the non-specificity of TEC should be improved to further advance cancer treatment.

Recently, studies of non-coding RNAs, including microRNAs, have attracted attention.^7^ Accumulating evidence indicates an important role of miRNAs in cancer progression.^8^ miRNAs regulate gene expression by promoting messenger RNA degradation or repressing messenger RNA translation. miRNAs are important regulators of various cellular processes, including development, differentiation, and signaling in cancer cells.^8^ Based on a deeper understanding of the role of miRNAs in cancer progression, miRNAs have been introduced as promising therapeutic targets for cancer treatment.^8^ Particularly, microRNA replacement therapy that restores downregulated cancer-specific microRNA using microRNA agonists/mimics is attracting attention as a next-generation molecular targeted therapy.^9^ Although studies regarding cancer cell microRNAs and the development of microRNA replacement therapy targeting cancer cells are ongoing, microRNAs associated with the phenotype of TECs have not been fully understood, and microRNA replacement therapy that targets TECs has not been studied yet.

Recent studies have shown that TECs have morphological, functional, and genetically different characteristics compared to vascular endothelial cells of healthy organs.^10^ In the study, we hypothesized that TEC-specific genes and microRNAs that are important to maintaining the phenotype of TECs exist, and regulating such genes specifically can inhibit tumor angiogenesis without affecting the vascular structure of healthy organs. This study aimed to establish a novel anti-angiogenic therapy targeting TEC using TEC-specific microRNAs.

## Results

### Current anti-angiogenic drugs affect not only tumor endothelial cells but also hepatic vasculatures

**Figure 1:**
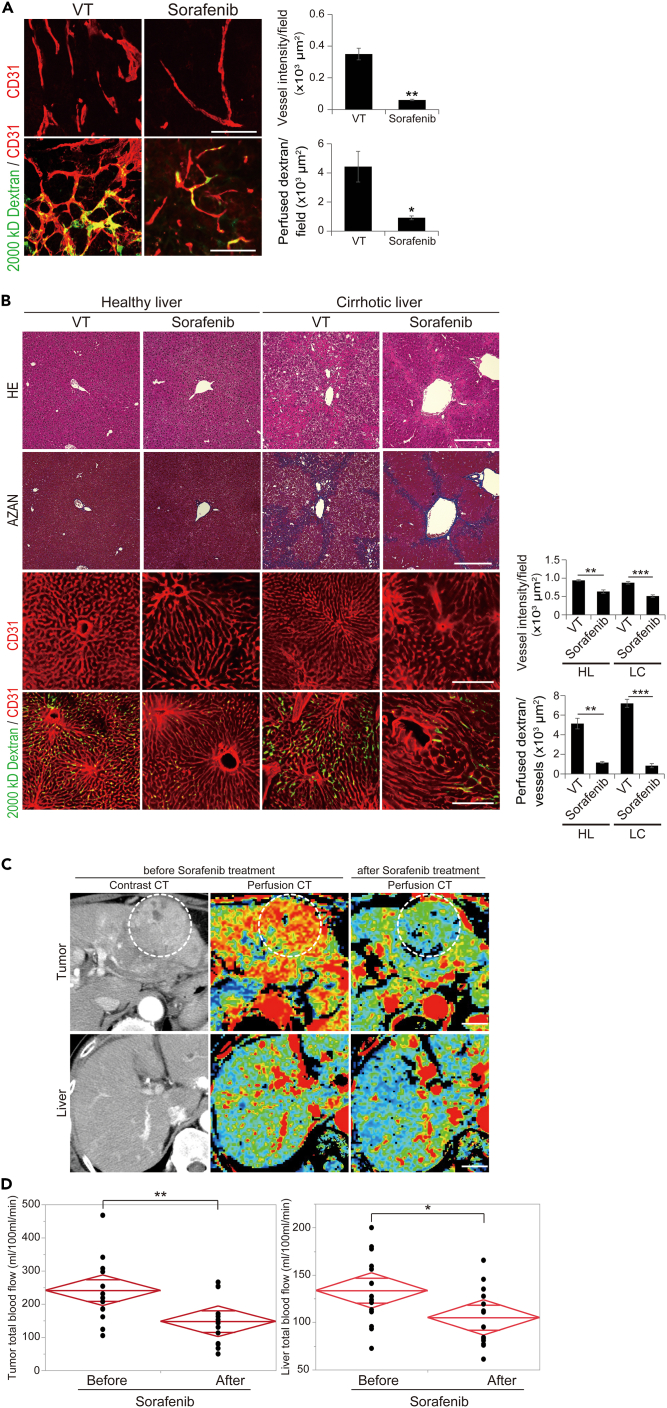
Evaluation of anti-angiogenic action for hepatic vasculatures in current anti-angiogenic drugs sorafenib (A) Whole-mount staining of tumor vessels and perfusion in HCC orthotopic mice treated with sorafenib (n = 6 random fields per group). Sorafenib significantly reduced vessel intensity (p < 0.01) and blood perfusion (p < 0.05). The scale bar represents 300 μm. (B) Whole-mount staining of tumor vessels and perfusion in healthy and cirrhotic livers treated with sorafenib (n = 5 random fields per group). Sorafenib significantly reduced blood perfusion in healthy (p < 0.01) and cirrhotic livers (p < 0.001). The scale bar represents 100 μm. (C) Perfusion CT in sorafenib-treated patients (n = 14). The scale bar represents 3 cm. (D) Quantification of tumor and hepatic blood flow in patients treated with sorafenib. Sorafenib significantly reduced blood flow in the tumor (p < 0.01) and liver (p < 0.001). HCC; hepatocellular carcinoma, CT; computed tomography.

Our previous studies revealed that the current AADs that target VEGF signaling inhibited tumor angiogenesis and affected the vasculature of healthy organs.^6^^,^^11^ We investigated whether AADs approved for treating HCC, such as sorafenib and lenvatinib, also affect hepatic vasculature. Sorafenib significantly inhibited tumor angiogenesis and reduced perfusion in a mouse HCC orthotopic model (Figure 1A). Sorafenib also reduced microvessel density and blood perfusion in healthy and cirrhotic livers (Figure 1B). This phenomenon was also observed in livers treated with lenvatinib (Figure S1). To increase the clinical relevance, we evaluated tumor and hepatic blood perfusion changes in 14 patients with HCC treated with sorafenib using perfusion computed tomography (CT) (Figures 1C and 1D). Table S1 shows the baseline clinical characteristics of the patients, and Figure S2 shows the overall survival and progression-free survival of the registered patients. As with the results of the mouse experiments, sorafenib significantly reduced the total blood flow (TBF) not only in the tumor and but also the surrounding liver even in the clinical practice (Figure 1C). Taken together, these data indicate that current AADs affect the hepatic vasculature and reduce blood flow in the liver.

### Tumor vessels are morphologically and functionally different from liver sinusoids

**Figure 2:**
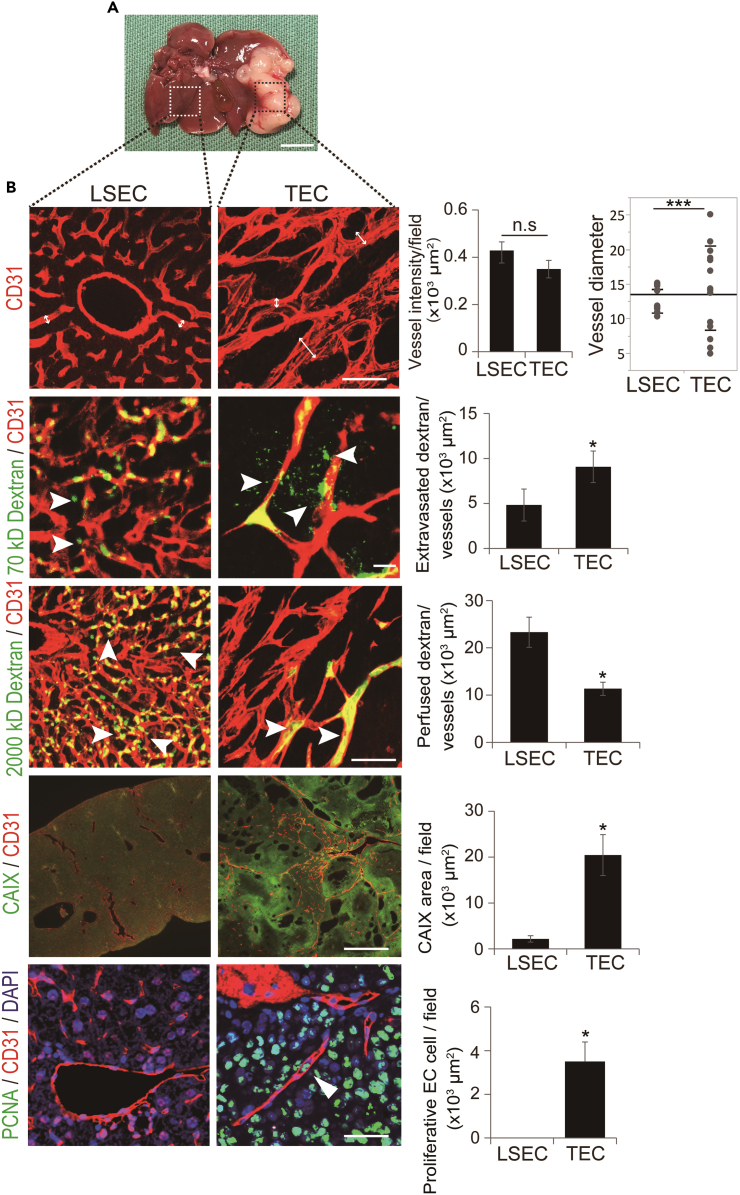
Evaluation of morphological and functional differences between TECs and LSECs (A) The macro-finding of the HCC orthotopic mouse model. (B) Whole-mount staining of tumor vessels and hepatic vasculature (scale bar: 300 μm). There was no significant difference in microvessel intensity between the two groups. There was a significant difference in the distribution of the vessel diameters (p < 0.001, n = 6 random fields per group). Whole-mount staining of 70 kD dextran and CD31 showed more leaked dextran (p < 0.05, n = 5 random fields per group, scale bar: 100 μm). Whole-mount staining of 2,000 kD dextran and CD31 showed fewer perfused vessels (p < 0.05, n = 5 random fields per group, scale bar: 100 μm). Immunohistochemical staining for CAIX and CD31 showed that the tumor had more hypoxia (p < 0.05, n = 6 random fields per group, scale bar: 20 μm). Immunohistochemical staining of PCNA and CD31 showed that proliferative endothelial cells were detected more in TEC (p < 0.05, n = 5 random fields per group, scale bar: 300 μm). TEC, tumor endothelial cell; LSEC, liver sinusoidal endothelial cell; HCC, hepatocellular carcinoma.

To understand the morphological and functional differences between TECs and hepatic endothelial cells, including LSEC, we created the HCC orthotopic mouse model (Figure 2A) and performed immunohistochemical staining. Although there was no significant difference in vessel intensity between TEC and LSEC, there was a significant difference in the distribution of vessel diameter between them, suggesting that TECs were significantly disorganized vessels (Figure 2B). In terms of function, tumor vessels showed leaky and less perfused vessels than the liver vasculature, followed by higher hypoxia in the tumor microenvironment (Figure 2B). Additionally, the number of proliferative endothelial cells was significantly higher in TEC than in LSEC (Figure 2B).

### Genetic differences between tumor endothelial cells and liver sinusoidal endothelial cells

**Figure 3:**
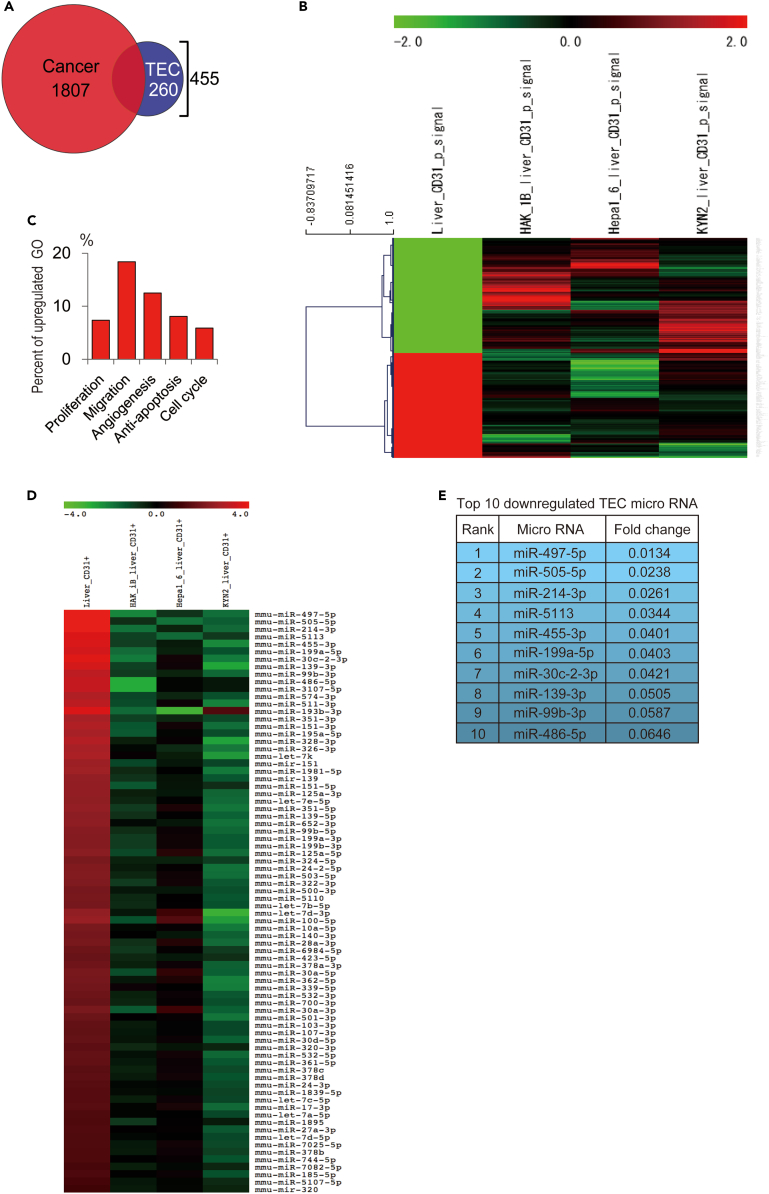
Evaluation of genetic difference between TECs and LSECs (A) Venn diagram of 260 TEC-specific genes. (B) Heatmap of TEC-specific genes. LSEC, TEC isolated from HAK1-B, Hepa1-6, and KYN-2 cells (n = 1 each). (C) Gene ontology analysis of TEC-specific gene expression. The upregulated genes showed proangiogenic features (proliferation, migration, angiogenesis, anti-apoptosis, and cell cycle). (D) Heatmap of TEC-specific miRNAs. LSEC, TEC isolated from HAK1-B, Hepa1-6, and KYN-2 cells (n = 1 each). (E) The top 10 downregulated TEC-specific microRNAs. TEC; tumor endothelial cell, LSEC; liver sinusoidal endothelial cell.

To shed light on the genetic differences between TEC and LSEC, we isolated the population of each vascular endothelial cell using magnetic beads. Fluorescence-activated cell sorting (FACS) analysis revealed that the TECs were isolated with over 80% purity (Figure S3). Compared to LSEC, 455 genes were significantly different between TECs and LSECs (Figure 3A). However, as cancer cell contamination is inevitable in the cell isolation method, we removed cancer cell-related genes from the 455 TEC-specific genes. Finally, we detected 260 TEC-specific genes that showed proliferative and pro-angiogenic features in the gene ontology analysis (Figures 3B and 3C). A microRNA array was used to detect TEC-specific miRNAs. We focused on downregulated miRNAs because the downregulation of TEC-specific miRNAs resulted in the upregulation of TEC-specific mRNAs (Figure 3D). The top 10 downregulated TEC-specific genes were detected using a microRNA array (Figure 3E).

### Detection of important microRNAs that are associated with the TEC phenotype

**Figure 4:**
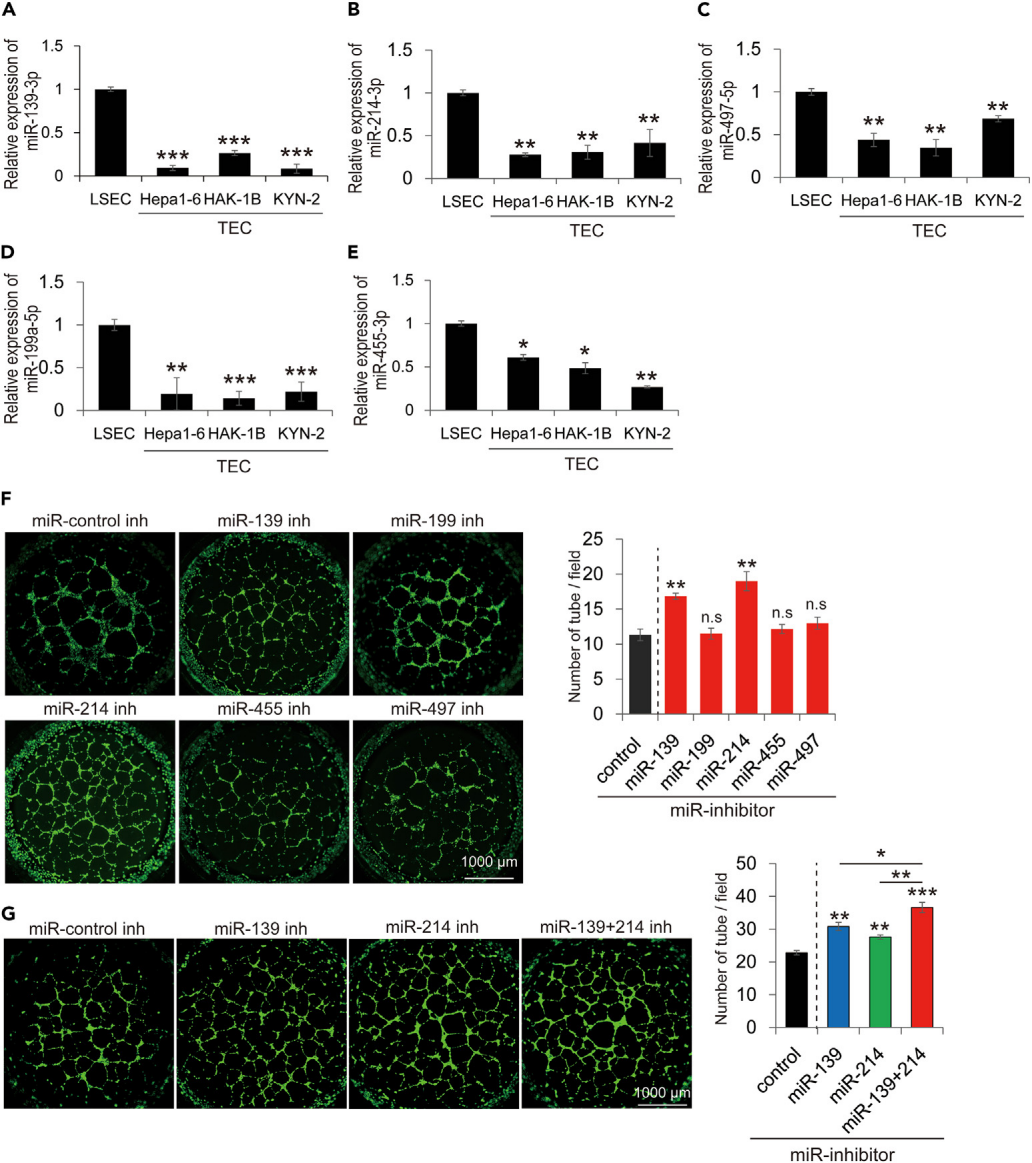
Evaluation of functional aspects in the detected TEC-specific microRNAs (A–E) A quantitative real-time PCR of the detected TEC-specific microRNAs. Among the top 10 downregulated miRNAs, miR-139-3p, 214-3p, 497-5p, 199a-5p, and 455-3p were significantly downregulated in TEC. (F) Tube formation assay using miRNA inhibitors (inh). Among the five candidate miRNAs, the miR-139 and 214 inhibitors significantly increased tube formation (n = 3). (G) Tube formation assay using miRNA inhibitors. The reduction of miR-139 and 214 significantly increased tube formation (n = 3). ∗p < 0.05, ∗∗p < 0.01, ∗∗∗p < 0.001, n.s.; not significant TEC; tumor endothelial cell, miR; microRNA.

Among the top 10 downregulated TEC-specific miRNAs, miR-139-3p, 214-3p, 199a-5p, 497-5p, and 455-3p were significantly downregulated in TECs (Figures 4A–4E and Figure S4). To assess whether these TEC-specific miRNAs are related to the TEC phenotype, tube formation assays using miRNA inhibitors were performed. In experiments involving miRNA inhibitors and mimics, we confirmed that the assay was appropriately conducted (Figure S5). Notably, the downregulation of miR-139-3p and 214-3p significantly increased tube formation (Figure 4F). The downregulation of these miRNAs simultaneously significantly increased tube formation compared with the downregulation of each microRNA (Figure 4G). These results suggest that miR-139-3p and miR-214-3p are important for achieving a TEC-specific phenotype in vascular endothelial cells.

### Upregulation of miR-139-3p and 214-3p using miR mimics inhibited tube formation

**Figure 5:**
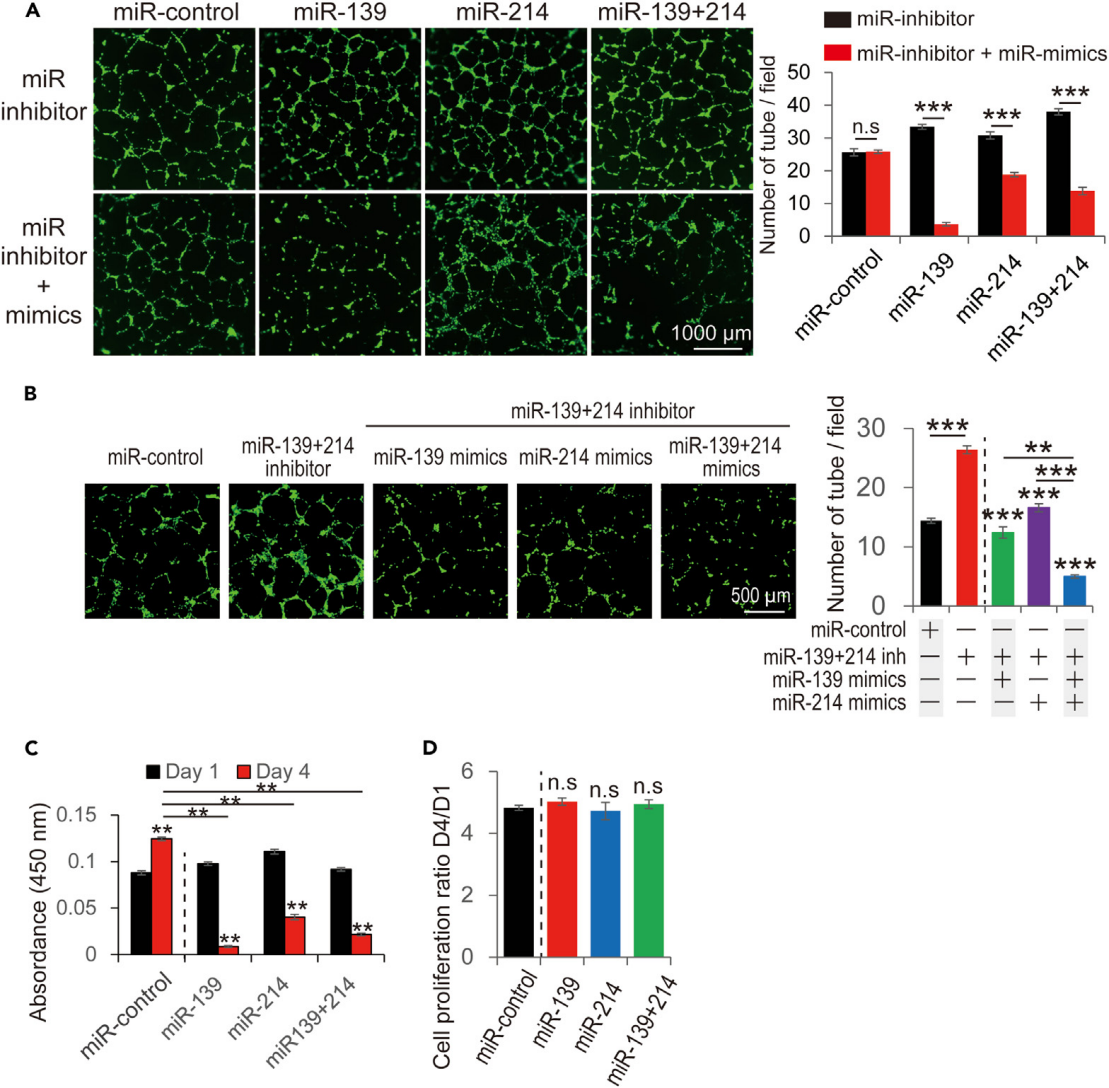
*In vitro* tube formation assay using the detected TEC-specific microRNAs mimics (A) Tube formation assay using miRNA mimics. After exposure to each or both miRNA inhibitors, each or both miRNA mimics were added to the culture media. The number of tubes formed was then counted (n = 3). (B) Tube formation assay using miRNA mimics. After exposure to both microRNA inhibitors, miRNA mimics were added to the culture media. (C) Cell proliferation assay of vascular endothelial cells (n = 6). (D) Cell proliferation assay of cancer cells (Hepa1-6, n = 6). ∗p < 0.05, ∗∗p < 0.01, ∗∗∗p < 0.001, n.s.; not significant TEC; tumor endothelial cell.

Experiments using microRNA mimics were performed to determine whether miR-139-3p and -214-3p are promising therapeutic targets. Upregulation of miR-139-3p and 214-3p using microRNA mimics significantly reduced tube formation (Figure S6). MicroRNA mimics were also reduced tube formation under the exposure of microRNA inhibitors (Figure 5A). As both miR-139-3p and -214-3p were downregulated in the isolated TECs, we evaluated the effects of the miRNA mimics on the downregulation of both miRNAs (Figure 5B). The upregulation of miR-139-3p and 214-3p significantly reduced the number of tubes formed under these conditions. Additionally, upregulation of these miRNAs inhibited cell proliferation in vascular endothelial cells (Figure 5C) but not in cancer cells (Figure 5D).

### TEC-specific microRNA agonist cocktail inhibited tumor growth through anti-angiogenesis without affecting hepatic vasculatures

**Figure 6:**
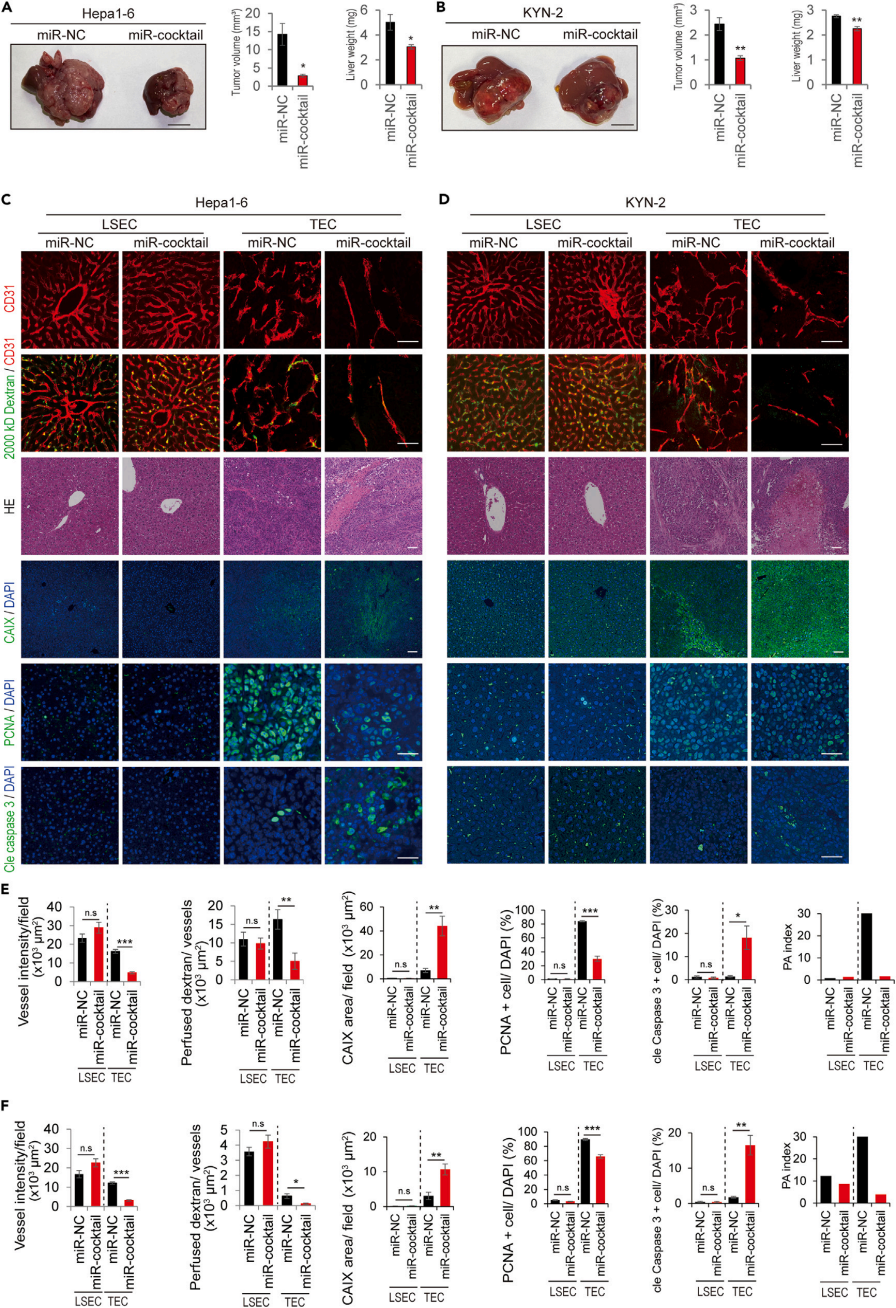
Evaluation of *in vivo* TEC-specific microRNA agonists cocktail for HCC orthotopic mouse models (A) Anti-tumor effect of TEC-specific microRNA agonist cocktail (miR cocktail) in Hepa1-6 orthotopic mouse model. The tumor volume (mm^3^) and whole liver weight (mg) were evaluated (n = 5). (B) Anti-tumor effect of the TEC-specific miR cocktail in the KYN-2 orthotopic mouse model. The tumor volume (mm^3^) and whole liver weight (mg) were evaluated (n = 5). (C and E) Immunohistochemical staining of each parameter in the Hepa1-6 orthotopic mouse model treated with the TEC-specific miR cocktail. Whole-mount staining of tumor vessels and hepatic vasculature (scale bar: 300 μm). Whole-mount staining of 2,000 kD dextran and CD31 (n = 5 random fields per group, scale bar: 100 μm). Immunohistochemical staining of CAIX (n = 6 random fields per group; scale bar: 100 μm). Immunohistochemical staining of PCNA (n = 5 random fields per group; scale bar: 300 μm). Immunohistochemical staining of cleaved caspase 3 (cle caspase 3, n = 5 random fields, scale bar: 300 μm). PA index was calculated using PCNA^+^ cells/cleaved caspase 3^+^ cells. (D and F) Immunohistochemical staining for each parameter in the KYN-2 orthotopic mouse model treated with a TEC-specific miR cocktail. Whole-mount staining of tumor vessels and hepatic vasculature (scale bar: 300 μm). Whole-mount staining of 2,000 kD dextran and CD31 (n = 5 random fields per group, scale bar: 100 μm). Immunohistochemical staining of CAIX (n = 6 random fields per group; scale bar: 100 μm). Immunohistochemical staining of PCNA (n = 5 random fields per group; scale bar: 300 μm). Immunohistochemical staining of cleaved caspase 3 (cle caspase 3, n = 5 random fields, scale bar: 300 μm). ∗p < 0.05, ∗∗p < 0.01, ∗∗∗p < 0.001, n.s.; not significant TEC; tumor endothelial cell, miR; microRNA, PA; proliferation-apoptosis.

To examine the effects of the detected TEC-specific miRNAs in treating HCC, the miRNA agonist cocktail (miR cocktail), including miR-139-3p and 214-3p mimics, was administered to the HCC orthotopic mouse models. A pilot study was performed using miR-negative and miR-positive controls (PC) to establish the assay. After injection, the administered miR-PC was detected in the tumor and TEC populations (Figures S7A and S7B). Moreover, the known target genes of miR-PC were significantly downregulated in the whole tumor and TEC populations (Figures S7C and S7D). We administered the miR cocktail to two orthotopic HCC mouse models based on the pilot study. Interestingly, the miR cocktail significantly inhibited tumor growth in both orthotopic HCC models (Figures 6A and 6B).

Changes in the tumor microenvironment due to the miR cocktail were evaluated using immunohistochemical staining (Figures 6C–6F). Tumor angiogenesis was significantly inhibited in tumors treated with the miR cocktail. Importantly, the miR cocktail did not decrease the vessel density in the hepatic vasculature. Significant hypoxia and necrosis in the tumor were detected following treatment with the miR cocktail. In addition, the miR cocktail treatment induced a significant reduction in cell proliferation, whereas an increase in cell apoptosis was observed only in the tumor, but not in the liver. These results suggest that the miR cocktail inhibits tumor growth by suppressing tumor angiogenesis without affecting the hepatic vasculature.

### TEC-specific microRNAs specifically inhibited vessel sprouting via the downregulation of vascular tip cells-related genes

**Figure 7:**
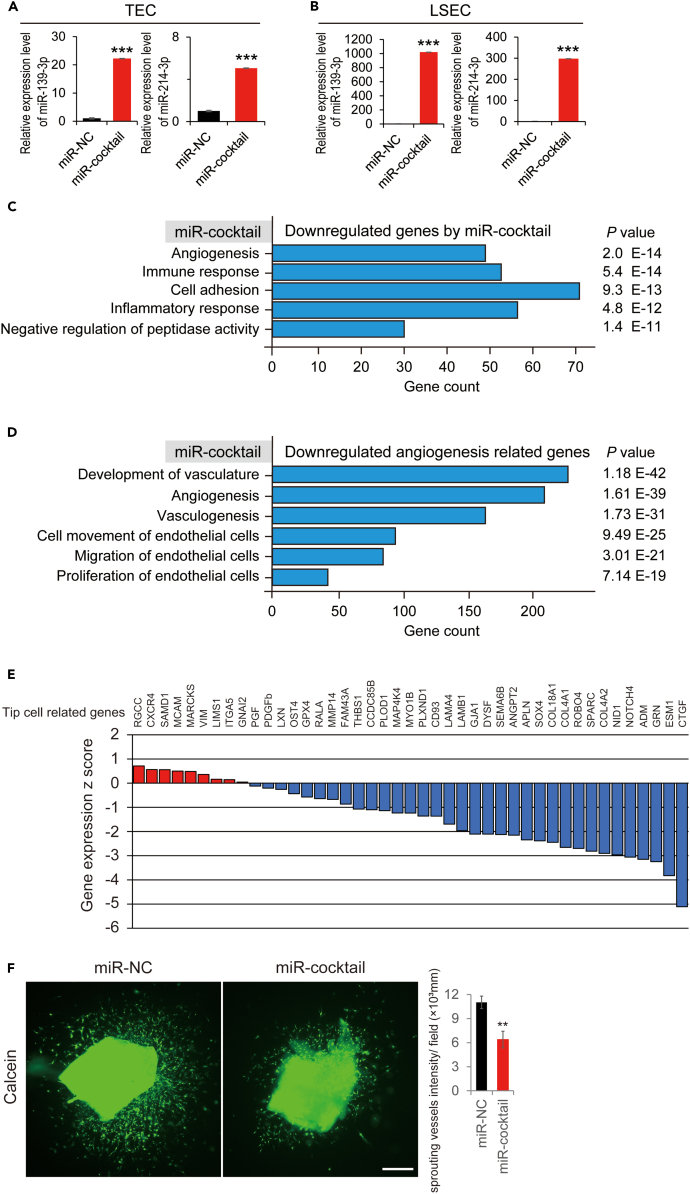
Evaluation of genetic change in TECs isolated from tumors treated with the TEC-specific microRNA agonists cocktail (A) Expression of TEC-specific microRNAs in TEC isolated from Hepa1-6 orthotopic mouse model treated with a TEC-specific miRNA agonist cocktail (miR cocktail). (B) Expression of TEC-specific microRNAs in LSEC isolated from Hepa1-6 orthotopic mouse model treated with the miR cocktail. (C) Gene ontology analysis of TECs treated with the miR cocktail (n = 1 in each group). (D) Angiogenesis-related gene ontology analysis of TECs treated with miR cocktail. (E) Waterfall plot of 46 tip-cell-related genes in TECs treated with the miR cocktail. Among them, the miR cocktail treatment downregulated 78% of the tip-cell-related genes. (F) Aortic ring assay in miR negative control (NC) and miR cocktail (n = 3). ∗p < 0.05, ∗∗p < 0.01, ∗∗∗p < 0.001, n.s.; not significant TEC, tumor endothelial cell; miR, microRNA; LSEC, liver sinusoidal endothelial cell.

These vascular endothelial cells were isolated to confirm whether the miR cocktail was transfected into TEC and LSEC. Notably, the miR-139-3p and 214-3p levels were significantly increased in both TEC and LSEC populations (Figures 7A and 7B). The expression gene array was performed, and gene ontology analysis was conducted to determine what happened in the tumor treated with the miR cocktail (Figure 7C). We found that angiogenesis-related genes were significantly downregulated by the miRNA cocktail. Particularly, various genes related to the dynamics of vascular endothelial cells, such as development, angiogenesis, vasculogenesis, cell movement, migration, and proliferation, were significantly reduced by the miR cocktail administration (Figure 7D). These results suggested that the miR cocktail significantly inhibited tumor angiogenesis via the downregulation of various angiogenesis-related genes. However, one remaining question was raised: there was no impact on the liver vasculature, regardless of transfection into LSEC (Figures 7A and 7B). To shed light on the reason why the miR cocktail only affects TECs, but not LSEC, we focused on vascular tip-cell-related genes. Goveia et al. reported approximately 43 vascular tip-cell-related genes in cancer, which were correlated with the vessel sprouting of TEC.^12^ Interestingly, among the 43 vascular tip-cell-related genes, 78% were downregulated by the miR cocktail treatment (Figure 7E). An aortic ring assay was performed to confirm whether the miR cocktail regulated vessel sprouting. The miR cocktail significantly reduced vessel sprouting (Figure 7F). Finally, the regulation of TEC-specific microRNAs detected in this study downregulated the genes related to vessel sprouting. Therefore, they could only affect tumor angiogenesis, but not the hepatic vasculature.

## Discussion

In this study, we identified two downregulated TEC-specific microRNAs, miR-139-3p and 214-3p, in orthotopic HCC mouse models. We further demonstrated that the upregulation of these miRNAs due to the miRNA agonist cocktail administration specifically inhibited tumor angiogenesis without affecting hepatic vasculature. These miRNAs regulate the genes related to vascular tip cell sprouting in tumor angiogenesis, which could separate the anti-angiogenic action between tumor vessels and healthy vessels.

Nowadays, the treatment for HCC has remarkably developed and many molecular targeted agents (MTAs) have been approved for treating HCC.^13^ The approved MTAs for HCC commonly inhibit tumor angiogenesis by blocking the VEGF signaling pathway.^2^ Additionally, anti-VEGF antibodies are also used in combination therapy with immune checkpoint inhibitors.^14^ Although current MTAs have proven survival benefits for patients with HCC, the incidence of adverse events and acquired resistance are becoming issues that must be solved. The incidence rate of adverse events of approved MTAs is >90% at any grade.^11^^,^^15^^,^^16^ The dose of MTAs must be reduced by the appearance of adverse events, which results in deterioration of the anti-tumor effect and induction of acquired resistance. Maintaining liver function is essential for treating HCC.^17^ However, as shown in this study, currently approved MTAs, such as sorafenib or lenvatinib, affect hepatic vasculature via VEGF blockade because VEGF is important for maintaining hepatic vasculature. This study showed that the TEC-specific microRNA cocktail did not affect the hepatic vasculature, which could be an ideal treatment in anti-angiogenic therapy for HCC.

Recent studies have shown that TEC has morphologically, functionally and genetically different characteristics compared to the vascular endothelial cells of healthy organs.^18^ Nanda et al. reported approximately 46 tumor endothelial markers (TEM), such as TEEM, TEM5, and TEM8.^19^ Hide et al. also reported that TECs are sensitive to growth factors and exhibit chromosomal abnormalities.^20^ These data indicated that TECs exhibit abnormalities and different phenotypes. Therapies targeting the TEC-specific gene have also been reported and have shown promising efficacy.^20^ However, studies on TEC-specific miRNAs have not yet been reported. This study is the first to report effective TEC-specific miRNAs in treating HCC, which has novelty and clinical significance from the point of view.

In this study, tumor angiogenesis was specifically inhibited by restoring/replacing the TEC-specific downregulated miRNAs. Such miRNA replacement therapy is an expected therapeutic concept for treating HCC using miRNA agonists.^9^^,^^21^^,^^22^^,^^23^ Diminished expression of tumor-specific miRNAs is correlated with tumor progression, invasion, and metastasis.^24^ miRNA replacement therapy aims to restore tumor suppressor miRNA function in tumor cells using synthetic miRNA mimics. Serej et al. and Orellana et al. demonstrated the efficacy of replacement therapy with miR Let-7 in treating cancer.^25^^,^^26^ This study was the first to demonstrate an effective microRNA replacement therapy that targets TEC. miRNAs significantly correlate with vascular development.^27^ This study revealed that microRNA replacement therapy targeting TEC-specific microRNAs could be a promising treatment for future molecular-targeted therapy.

This study found two important miRNAs that are essential for achieving the TEC phenotype. The first is miR-139-3p. Chi et al. reported that miR-139-3p downregulation was correlated with angiogenesis by regulating the expression of the S100A14 pathway.^28^ miR-139-3p is also associated with cancer cell proliferation.^29^ The second factor was miR-214-3p expression. Xiao et al. reported that miR-214-3p is correlated with endothelial migration and angiogenesis by regulating TrkB/ShcB expression.^30^ In this study, we found that these TEC-specific miRNAs significantly regulate the expression of vascular tip cell-related genes. Generally, vascular sprouting occurs during the fetal period or cancer progression.^31^ As the phenomenon does not generally occur in quiescent vascular cells in healthy organs, the treatment using the TEC-specific microRNAs detected in the study can only inhibit tumor angiogenesis with specificity, without affecting quiescent vascular cells in healthy organs.

In conclusion, we identified two downregulated TEC-specific miRNAs in orthotopic HCC mouse models. Furthermore, we demonstrated that the TEC-specific miRNA cocktail significantly inhibited tumor growth by inhibiting tumor angiogenesis without affecting hepatic vasculature. TEC-specific miRNA replacement therapy that targets only tumor-sprouting vessels can be a novel therapeutic concept and a promising treatment for patients with HCC.

### Limitations of the study

This study found promising TEC-specific miRNAs that could be therapeutic targets in treating HCC. However, this study had some limitations. Although we found that these miRNAs significantly inhibited tumor angiogenesis by downregulating vascular tip cell-related genes, we could not identify the genes specifically binding to the detected TEC-specific microRNAs. Further studies are needed to identify the target genes. And we detected TEC-specific miRNAs only from downregulated genes isolated from TEC because the concept of the miRNA replacement therapy is to compensate the lacking miRNA. However, important miRNAs could also exist on the upregulated miRNAs. Therefore, further experiments are needed in the study. Moreover, its clinical relevance should be studied in future research. Whether TEC-specific microRNAs are detected in TECs isolated from human HCC samples and whether the regulation of these miRNAs is functional even for patients with HCC should be studied. However, the concepts identified in this study are important. The issue regarding the non-specificity of the targeted molecules for TEC in current AADs should be solved for further development in the field of anti-angiogenic treatment. The findings of the study showed promising outcomes from the point of view.

## STAR★Methods

### Key resources table

**Table:** 

REAGENT or RESOURCE	SOURCE	IDENTIFIER
**Antibodies**		

Anti-CD31 antibody	R&D Systemc Inc	AF-3628
Donkey anti-goat antibody	Abcam	ab150130
Lysine-fixable tetramethyl rhodamine dextran 70kDa	Invitrogen	D1818
Lysine-fixable tetramethyl rhodamine dextran 2000kDa	Invitrogen	D7139
Anti-Carbonic anhydrase IX antibody	Abcam	ab184006
Anti-cleaved caspase 3 antibody	Cell signaling	#9661
Anti-PCNA antibody	Santa Cruz	#sc-7907
Goat anti-rabbit antibody	Invitrogen	#A11034
anti-CD31 antibody	Miltenyi Biotech	130-097-418

**Critical commercial assays**		

Angiogenesis assay Kit	Ibidi	#81505

**Experimental models: cell lines**		

Hepa1-6	ATCC	CRL-1830
HAK1-B	Our facility	Hepatology. 1993 Aug;18(2):320-7.
KYN-2	Our facility	Acta Pathol Jpn. 1988 Aug;38(8):953-66.

**Deposited data**		

Sequencing Data	National Center for Biotechnology Information	GSE250147

**Oligonucleotides**		

miR-139-3p inhibitor and mimics	Bioneer	MI0000261
miR-negative control	Bioneer	SMC-4001
miR-214-3p inhibitor and mimics	Bioneer	MI0000290
miR-199a inhibitor	Bioneer	MI0000242
miR-455-3p inhibitor	Bioneer	MI0003513
miR-497-5p	Bioneer	MI0003138
miR-negative control	Thermo Fisher	4464059
miR--1 positive control	Thermo Fisher	4464062
miR-139-3p	Thermo Fisher	MC12430
miR-214-3p	Thermo Fisher	MC12124

**Software and algorithms**		

DAVID	National Institutes of Health	DAVID Functional Annotation Bioinformatics Microarray Analysis (ncifcrf.gov)

### Resource availability

#### Lead contact

Further information regarding this manuscript and requests should be directed to the lead contact, Hideki Iwamoto, MD., PhD. (iwamoto_hideki@med.kurume-u.ac.jp)

#### Materials availability

This study did not generate any new materials.

#### Data and code availability

• Deidentified final results supporting this study are available for research purposes upon reasonable written request to the corresponding author. Access to such data is available from the date of publication and requires a Data Access Agreement, which is examined and approved by the ethics committees who approved this research.
• This paper does not report original code.
• Any additional information required to reanalyze the data reported in this paper is available from the lead contact upon request.

### Experimental model and study participant details

#### Ethics statement

All animal experiments were approved by the ethical committee of the Kurume University School of Medicine (2021-185). The study regarding perfusion CT was approved by the Ethical Committee of Kindai University (23-105) and conducted per the Declaration of Helsinki. Written informed consent for perfusion CT was obtained from each patient.

#### Establishment of the hepatoma orthotopic mouse model

To establish the hepatoma orthotopic mouse models, 1.0×10^6^ Hepa1-6, HAK-1B, and KYN-2 cells were suspended in 50 mL solution (25 μL phosphate-buffered saline; PBS + 25 μL Matrigel matrix, Corning, NY, USA) and orthotopically inoculated into the left lobe of the mouse liver. The peritoneum and skin were surgically sutured under isoflurane anesthesia (Wako Pure Chemical Industries, Tokyo, Japan).

To assess the anti-angiogenic effects of sorafenib and lenvatinib on tumor angiogenesis, we created a Hepa1-6 hepatoma orthotopic mouse model. 30 mg/kg/mouse of sorafenib (ChemScene, Monmouth Junction, NJ, USA) or 10 mg/kg mouse lenvatinib (ChemScene) was administered once daily during daylight hours.

To assess the effect of AADs on the vascular structure of the liver, we treated healthy mice and mice that developed liver cirrhosis with sorafenib. The healthy mice were treated with lenvatinib. Liver cirrhosis was induced by carbon tetrachloride administration (CCl_4_; Wako Pure Chemical Industries; 1 μL/g body weight in olive oil). CCL_4_ was intraperitoneally injected into mice under inhalational anesthesia using 3% isoflurane twice a week until week 8.

To assess the effect of *in vivo* miRNA agonist cocktail including miR-139-3p and 214-3p mimics, Hepa1-6 and KYN-2 hepatoma orthotopic mouse models were established. The negative control (4464059), miR-1 positive control (4464062), miR-139-3p (MC12430), and 214-3p mimic (MC12124) were purchased from Thermo Fisher (Ambion mirVana). The *in vivo* miR agonists (2 nmol) were injected twice a week via the tail vein at 10 ng/mouse with Invivofectamine 3.0 (Thermo Fisher), following the manufacturer’s instructions. All treatments were continued for two weeks, after which the mice were sacrificed. Tumor volume was calculated by measuring tumor diameter from the surface (Tumor volume= length×width×width/2).

#### Patients

Perfusion CT was conducted for patients with HCC treated with sorafenib in the Kinki University Hospital between 2009 and 2010.

### Method details

#### Cell lines and animals

Mouse hepatoma cell line Hepa1-6 was purchased from American Type Culture Collection (ATCC; VA, USA). The human hepatoma cell lines, HAK-1B and KYN-2, were originally established and maintained at our institute. Both human and mouse tumor cell lines were grown and maintained in DMEM (Gibco by Invitrogen Cell Culture Co., Auckland, New Zealand) containing 10% (v/v) heat-inactivated fetal bovine serum (FBS; Biowest, Nuaille, France) and 100 U/mL Penicillin-Streptomycin (Nacalai Tesque, Kyoto, Japan) in a humidified atmosphere containing 5% CO_2_ at 37°C. Human umbilical vein endothelial cells and mouse tumor vascular endothelial cells 2H-11 were purchased from ATCC and maintained in a complete EGM-2 medium (Lonza, Walkersville, MD, USA).

Female 6-week-old C57BL/6 or nude mice (BALB/c nu/nu) were purchased from Kyudo KK (Fukuoka, Japan). We chose female mice to avoid “fighting” during the experiments. The mice were caged in groups of six or fewer mice per cage at the Kurume University School of Medicine animal facility. The mice were sacrificed via cervical dislocation after anesthesia with isoflurane. All animal experiments were performed using five–six mice per group.

#### Perfusion CT

CT acquisition for hepatic perfusion was performed using a single-source 64-channel multidetector CT scanner (Discovery CT750 HD; GE Healthcare, Milwaukee, WI, USA). The patients underwent a dynamic perfusion CT scan in addition to the routinely requested CT scan. Perfusion CT was performed per the manufacturer’s recommendations. The iodinated contrast material iohexol (Omnipaque 300 mg/ml, Daiichi-Sankyo Pharma, Tokyo, Japan) was injected intravenously at 4–5 mL/s at an iodine dosage of 600 mg/kg, tailored by patient weight. Dynamic scanning of a 75-mm liver section was conducted under 120 kV, 70 mA, and 0.4 sec of gantry rotation speed by using an axial shuttle mode, in which the scanner table was joggled 42 times between 2 adjacent 40-mm sections (there was 5-mm overlap between the 2 table positions). The total duration of the imaging study was roughly 2.1 min, and the patient breathed freely throughout the study. Each set of reconstructed perfusion images was manually analyzed using a CT Perfusion software (GE Healthcare) to generate total blood flow (TBF) using a model-based deconvolution algorithm in which the Region of Interest can be positioned in the tumor and the surrounding liver.

#### Whole-mount staining

Whole-mount staining was performed as described previously.^32^^,^^33^ Briefly, tissues were cut into thin slices, fixed in 4% paraformaldehyde overnight, and then exposed to 20 μg/mL proteinase K. Thereafter, the tissues were incubated with a goat anti-CD31 antibody (R&D Systems Inc., Minneapolis, MN, USA, Cat. No. AF-3628) overnight at 4°C, followed by staining with a donkey anti-goat secondary antibody (Abcam, Tokyo, Japan) for 2 h at room temperature. To assess vascular permeability and leakiness, lysine-fixable tetramethyl rhodamine dextran with a molecular weight of 70 kDa or 2000 kDa (Invitrogen) was injected into the tail vein. The mice were sacrificed five minutes after injection with 2000 kD and 15 min after injection with 70 kD. The slides were mounted and examined under a microscope (Keyence Corporation, Osaka, Japan). We scanned five thin sections at 3-μm distances from each sample and projected three-dimensional images of each tissue sample. Quantitative analyses of at least six different sections were performed using the Adobe Photoshop CS software (Adobe Systems, Tokyo, Japan).

#### Immunohistochemical staining

Immunohistochemical staining was performed as previously described. Briefly, paraffin-embedded tumor tissue sections (5 μm thick) were boiled for 30 min in a high-pH target retrieval solution for antigen retrieval and subsequently incubated with primary antibodies followed by secondary antibodies. The antibodies used are listed in Table S2. Nuclei were counterstained with DAPT (Vector Laboratories, Inc., Burlingame, CA, USA, # H-1200).

#### Isolation of CD31^+^ cells from tumor tissues by magnetic beads

Fresh tumor tissues were cut into small pieces in ice-cold PBS and digested in PBS containing 0.1% collagenase II (Worthington Biochemical Corporation, NJ, USA, CLS2 LS004174) at 37°C for 30 min with gentle pipetting to perform single-cell digestion. After digestion, cells were washed with Hank’s buffer (HBSS, Thermo Fisher, Gibco 14025-092) and blocked with FcR Blocking Reagent for 10 min (Miltenyi Biotech, Tokyo, Japan, 1130-023-575) and resuspended in 1 mL magnetic-activated cell sorting (MACS) buffer (Miltenyi Biotech 130-091-222), and anti-mouse CD31 MicroBeads (Miltenyi Biotech 130-097-418) were used for magnetic labeling. After washing, positive and negative cells were sorted using a MACS column and magnetic MACS separators, according to the manufacturer’s protocol.

#### Gene expression array

LSECs were isolated from the livers of mice, and TECs were obtained from tumors established by the hepatoma cell lines Hepa1-6, Huh-7, and KYN-2 to detect the difference in gene expression profiles between LSEC and TEC. TECs were isolated from tumors treated with the negative control and microRNA-agonist cocktail to evaluate the change in gene expression profiles by the microRNA-agonist cocktail. RNA was extracted using NucleoSpin miRNA (Macherey-Nagel, PA, USA). Gene expression microarray was analyzed using a Cell Innovator (Fukuoka, Japan). The cRNA was amplified, labeled, and hybridized to a 60 K Agilent 60-mer oligo microarray per the manufacturer’s instructions. All hybridized microarray slides were scanned using an Agilent scanner. Relative hybridization intensities and background hybridization values were calculated using the Agilent Feature Extraction Software (9.5.1.1). Functional analysis of the changing gene expression was performed using the Kyoto Encyclopedia of Genes and Genomes (KEGG) database on the DAVID system. Raw microarray data were submitted to Gene Expression Omnibus GEO in NCBI.

#### Data analysis and filter criteria

Regarding data analysis and filter criteria, raw signal intensities and flags for each probe were calculated from hybridization intensities (gProcessedSignal) and spot information (gIsSaturated), according to the procedures recommended by Agilent. (Flag criteria in GeneSpring Software. Absent (A): “Feature is not positive and significant” and “Feature is not above background”. Marginal (M): “Feature is not Uniform,” “Feature is Saturated,” and “Feature is a population outlier”. Present (P): others). Moreover, the raw signal intensities of the two samples were log2-transformed and normalized by a quantile algorithm with the ‘preprocessCore’ library package^34^ on Bioconductor software.^35^ We selected probes that call the ‘P’ flag at least one sample, excluding lincRNA probes. To identify up-or downregulated genes, we calculated Z-scores^36^ and ratios (non-log scaled fold-change) from the normalized signal intensities of each probe for comparison between control and experimental samples. Then, we established criteria for regulated genes: (upregulated genes) Z-score ≥ 2.0, ratio ≥ 1.5-fold, (downregulated genes) Z-score ≤ -2.0, and ratio ≤ 0.66.

#### MicroRNA array

LSECs were isolated from the livers of mice, and TECs were obtained from tumors established by each hepatoma cell line, Hepa1-6, Huh-7, and KYN-2 and the differences in their miRNA profiles were evaluated. A total of 100 ng of total RNA from each sample was labeled using the FlashTag™ Biotin HSR RNA Labeling Kit and hybridized to an Affymetrix GeneChip® miRNA 4.0 Array according to the manufacturer’s instructions. All the hybridized microarrays were scanned using an Affymetrix scanner. Relative hybridization intensities and background hybridization values were calculated using the Affymetrix Expression Console™.

We processed the raw CEL files for gene-level analysis with median polish summarization and quantile normalization using the Affymetrix® Transcriptome Analysis Console Software, and normalized intensity values were obtained. To identify up-or downregulated genes, we calculated ratios (non-log scaled fold-change) from the normalized intensities of each gene for comparisons between control and experimental samples. Then, we established criteria for regulated genes: (upregulated genes) ratio ≥ 2.0-fold, (downregulated genes) ratio ≤ 0.5.

#### Reverse transcription-quantitative real-time PCR

Real-time PCR was performed as described in our previous report. Briefly, RNA and microRNA were extracted using a NucleoSpin miRNA (MACHEREY-NAGEL), and then reverse transcribed into cDNA. Quantitative real-time PCR was performed to detect the messenger RNA and microRNAs. *Glyceraldehyde-3-phosphate dehydrogenase (Gapdh)* was used as the housekeeping gene. U6 was used as a housekeeping miRNA. The primers were purchased from TaqMan™ (Applied Biosystems, CA, USA) and were as follows: *Gapdh*, Mm99999915_g1, Twf1, Mm00725968_s1, U6 snRNA (#001973), mmu-miR-1a-1 (mmu482914_mir), mmu-miR-497-5p (mmu482607_mir), mmu-miR-505-5p (mmu481200_mir), mmu-miR-214-3p (mmu481652_mir), mmu-miR-199a (mmu480983_mir), mmu-miR-30c-2 (mmu479401_mir), mmu-miR-139-3p (mmu480929_mir), mmu-miR-99b (mmu481283_mir), mmu-miR-486 (mmu483002_mir), mmu-miR-455-3p (mmu482767_mir), and mmu-miR5113 (mmu481884_mir).

#### *In vitro* transfection of microRNA inhibitor and mimics

Vascular endothelial cells were seeded on 6 well plates. Synthetic miRNA inhibitors and mimics were purchased from Bioneer (Oakland, CA, USA). The negative control (SMC-4001), miR-139-3p inhibitor and mimics (MI0000261), miR-214-3p inhibitor and mimics (MI0000290), miR-199a inhibitor (MI0000242), miR-455-3p inhibitor (MI0003513), and miR-497-5p (MI0003138) were used at a final concentration of 50 nM. The cells were transfected using Lipofectamine RNAiMAX reagent (Thermo Fisher, 13778030). After overnight transfection, cells were used for each experiment.

#### Tube formation assay

Tube formation assays were performed using a μ-Slide Angiogenesis Kit (#81506. Ibidi GmbH, Gräfelfing, Germany) according to the manufacturer’s protocol. EGM-2, without hydrocortisone, was used in the assay. On day 1, transfection with miRNA inhibitors or mimics was performed overnight. On day 2, cells were seeded and cultured overnight. On day 3, the cells were stained with Calcein AM Solution (1:1000, #NV011, Fujifilm, Tokyo, Japan) for 1 h. The tubes were counted using the Adobe Photoshop CS software (Adobe Systems).

#### Cell proliferation assay

Cells were seeded onto 96-well plates at a density of 1000 cells/well in sextuplicates and incubated in 100 mL of culture medium for 24 h. After overnight incubation at 37°C in a serum-free medium, the medium was changed. Cell proliferation at various time points was determined using Cell Count Reagent SF (Nacalai Tesque), according to the manufacturer’s protocol.

#### Aortic ring assay

The aortic ring assay was performed as described previously.^37^ Thoracic aortae were dissected from C57/BL6 mice, cleaned, and cut into rings. The cells were then serum-starved overnight. During serum starvation, the rings were transfected with a miR agonist cocktail or negative control. The next day, rings were embedded in the following extracellular matrices: rat-tail collagen type I (Millipore, Tokyo, Japan, 08-115); Medium 199 10× phenol red-free (Thermo Fisher); 140 mM NaHCO_3_, 1 M NaOH, and filtered and autoclaved water. After 24 h, immunofluorescence staining was performed using Isolectin GS-IB4 AF488 conjugate (Thermo Fisher, I21411). The samples were then scanned using a fluorescence microscope (Keyence). Quantitative analyses were performed using Adobe Photoshop CS software program (Adobe system).

### Quantification and statistical analysis

#### Statistical analysis

All statistical analyses were performed using the JMP statistical analysis software (JMP Pro version 16, SAS Institute Japan Inc., Tokyo, Japan). Overall survival and progression-free survival were calculated using the Kaplan-Meier method and analyzed using the log-rank test. All experimental data are expressed as mean ± SD. Between-group comparisons were performed using the Mann-Whitney U test, Kruskal-Wallis test, and nonparametric analysis of variance. If the one-way analysis of variance was significant, differences between individual groups were analyzed using the Fisher’s least significant difference test. P<0.05 was considered statistically significant.
