# Supplementary material for: A tumor endothelial cell-specific microRNA replacement therapy for hepatocellular carcinoma
Source: iScience. 2024 Jan 4;27(2):108797. doi: 10.1016/j.isci.2024.108797 (PMC10831275; doi:10.1016/j.isci.2024.108797)

iScience, Volume *27*

Supplemental information

A tumor endothelial cell-specific microRNA replacement therapy for hepatocellular carcinoma

Hideki Iwamoto, Hiroyuki Suzuki, Atsutaka Masuda, Takahiko Sakaue, Toru Nakamura, Toshimitsu Tanaka, Miwa Sakai, Yasuko Imamura, Hirohisa Yano, Takuji Torimura, Hironori Koga, Kaori Yasuda, Masakatsu Tsurusaki, Takahiro Seki, and Takumi Kawaguchi

Table S1. Baseline clinical and tumor characteristics of the patients, related to Figure 1.

Table S2. List of antibodies used for immunohistochemistry, related to STAR Methods.

Figure S1. Immunohistochemical staining of CD31, related to Figure 1.

Figure S2. Overall survival and progression-free survival of patients treated with sorafenib, related to Figure 1.

Figure S3. Purity of Isolated CD31-positive cells, related to Figure 3.

Figure S4. Quantitative real-time PCR of five microRNAs among the top 10 downregulated microRNAs, related to Figure 4.

Figure S5. Transfection efficiency of microRNA (miRNA) inhibitor/mimics, related to Figure 4.

Figure S6. Tube formation assay using miRNA mimics, related to Figure 5

Figure S7. The pilot experiment of in vivo microRNA negative (NC) and positive control (PC) for the HCC, related to Figure 6.

Table S1. Baseline clinical and tumor characteristics of the patients, related to Figure 1.

Characteristic	All patients
N	14
Age (years)	70.5 (54–86)
Sex (female/male)	2/12
Etiology (HBV/HCV/nonBnonC)	3/9/2
Child-Pugh class (A/B)	4/10
BCLC stage (B/C)	8/6
Tumor size (mm)	45 (20–101)
AFP (ng/mL)	461 (4–68,442)
DCP (mAU/mL)	3,395 (11–72,050)
Initial dose (mg/day) 800/400	12/2
Duration of sorafenib treatment (months)	5.4 (0.37–42.7)

Data are expressed as median (range), or number.

Abbreviations: BCLC stage, Barcelona Clinic Liver Cancer stage; AFP, α-fetoprotein; DCP, des-γ-carboxy prothrombin

Table S2. List of antibodies used for immunohistochemistry, related to STAR Methods.

Antibody	Source	Dilution
Carbonic anhydrase IX (rabbit pAb)	#ab184006; Abcam	1:500
CD31 (goat pAb)	#AF3628; R&D Systems	1:200
Cleaved caspase-3 (rabbit mAb)	#9661; Cell Signaling Technology	1:400
PCNA (rabbit pAb)	#sc-7907; Santa Cruz Biotechnology	1:100
Alexa Fluor™ 555 (donkey anti-goat IgG)	#ab150130; Abcam	1:200
Alexa Fluor™ 488 (goat anti-rabbit IgG)	#A11034; Thermo Fisher Scientific	1:200

Abbreviations: pAb, polyclonal antibody; mAb, monoclonal antibody; PCNA, proliferating cell nuclear antigen

Figure S1. Immunohistochemical staining of CD31, related to Figure 1.

Immunohistochemical staining of CD31 in healthy liver treated with lenvatinib (*n*=6 random fields per group). The scale bar represents 100 μm.

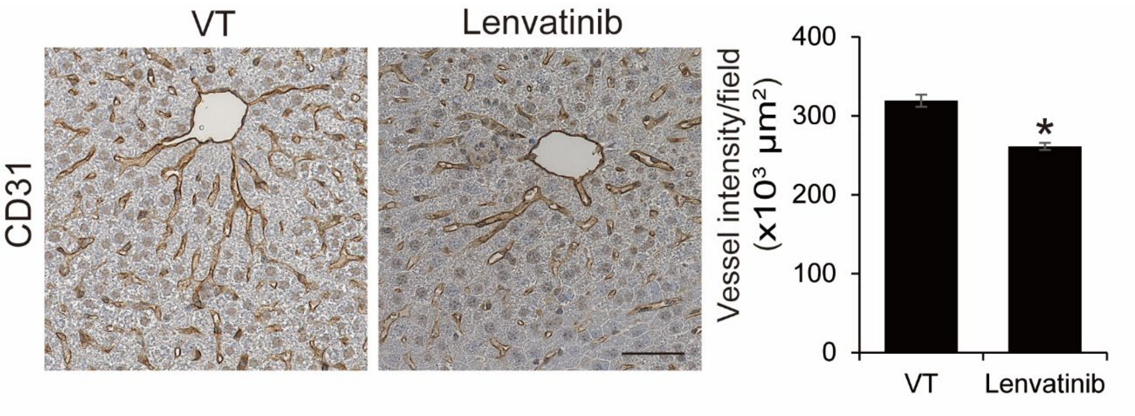

Figure S2. Overall survival and progression-free survival of patients treated with sorafenib, related to Figure 1.

A) Overall survival curves of patients with hepatocellular carcinoma (HCC) treated with sorafenib. The median survival time was 14.2 months.

B) Progression-free survival curve of patients with HCC treated with sorafenib. The median progression-free survival time was 7.6 months.

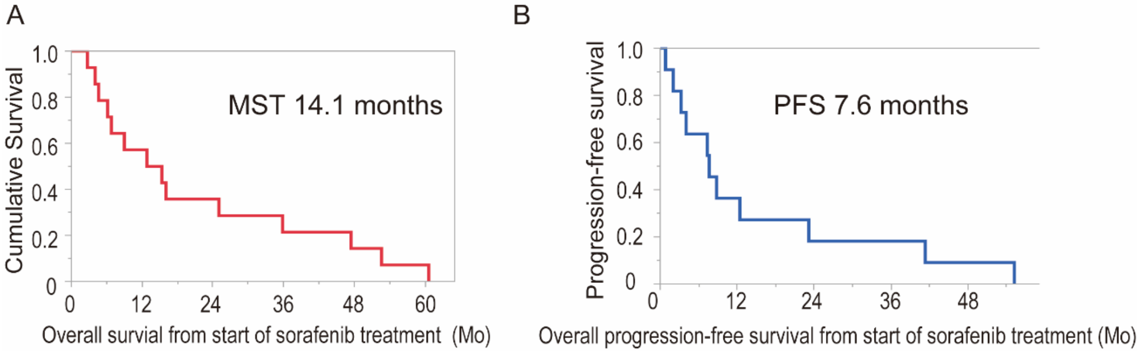

Figure S3. Purity of Isolated CD31-positive cells, related to Figure 3.

A) Purity of the isolated liver sinusoidal endothelial cell (LSEC)

B) Purity of the tumor endothelial cell (TEC) isolated from Hepa1-6 orthotopic mouse model

C) Purity of the TEC isolated from HAK1-B orthotopic mouse model

D) Purity of the TEC isolated from KYN-2 orthotopic mouse model

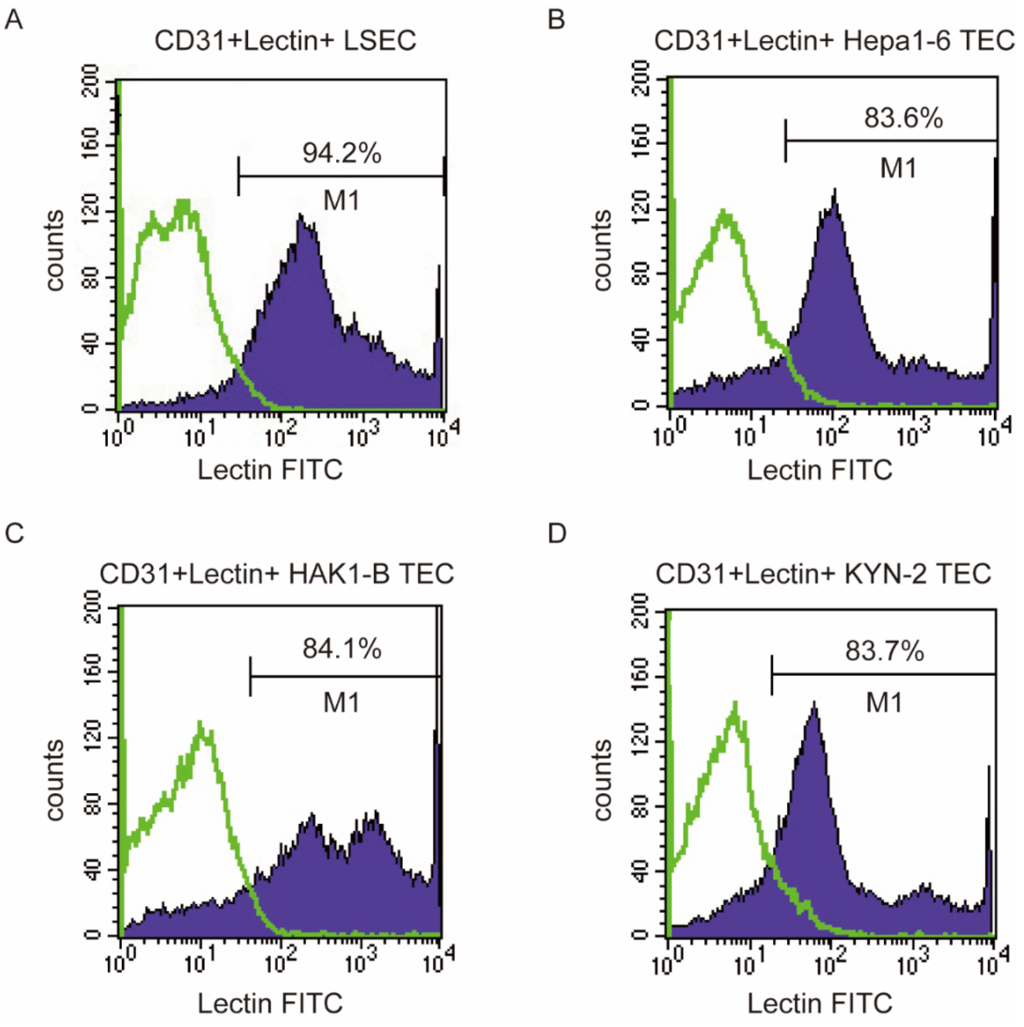

Figure S4. Quantitative real-time PCR of five microRNAs among the top 10 downregulated microRNAs, related to Figure 4.

There were no significant differences in miR-99b, 30c-2, 505-5p, 5113, and 486. The expression of U6 is used as the housekeeping microRNA. * p<0.05, ** p<0.01, n.s; not significant

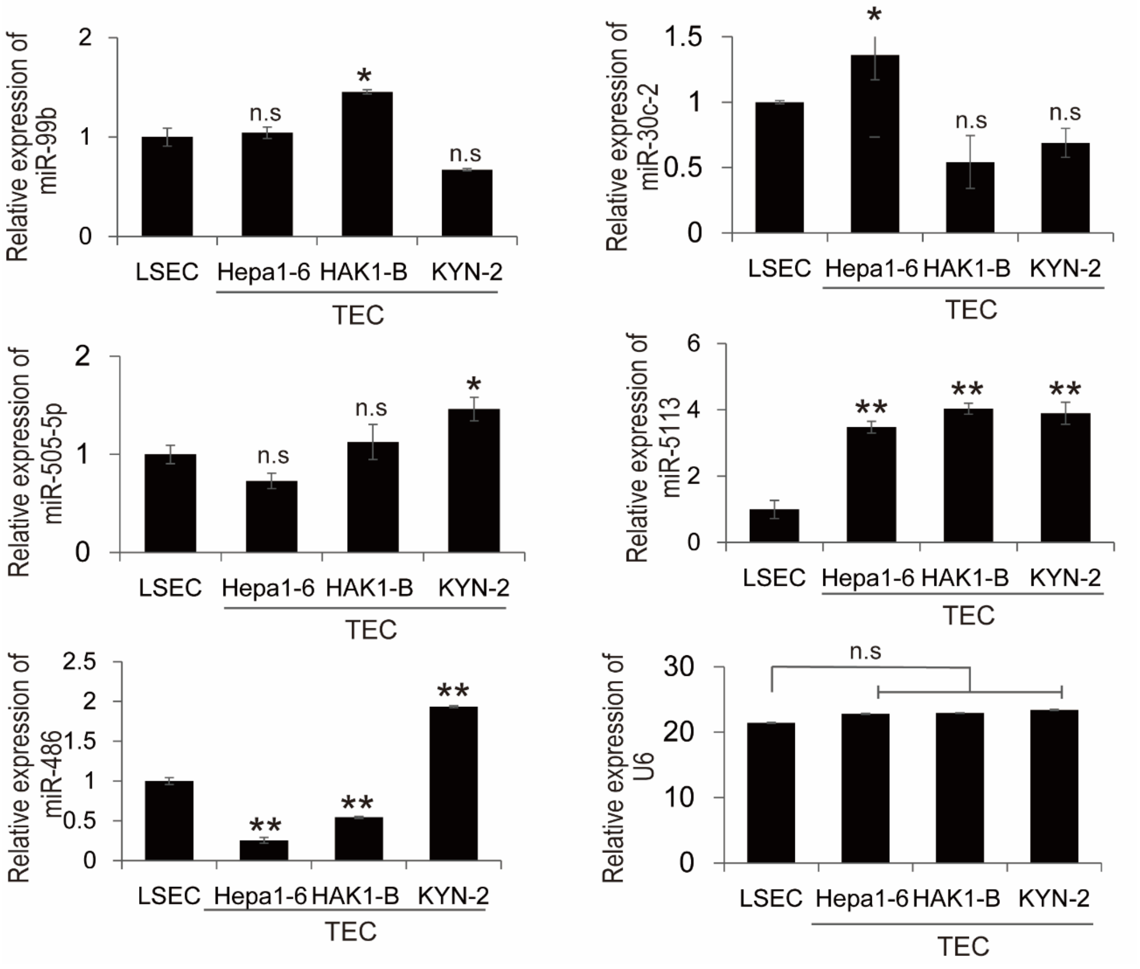

Figure S5. Transfection efficiency of microRNA (miRNA) inhibitor/mimics, related to Figure 4.

The GFP-positive cells were seen in more than 90% of the cells.

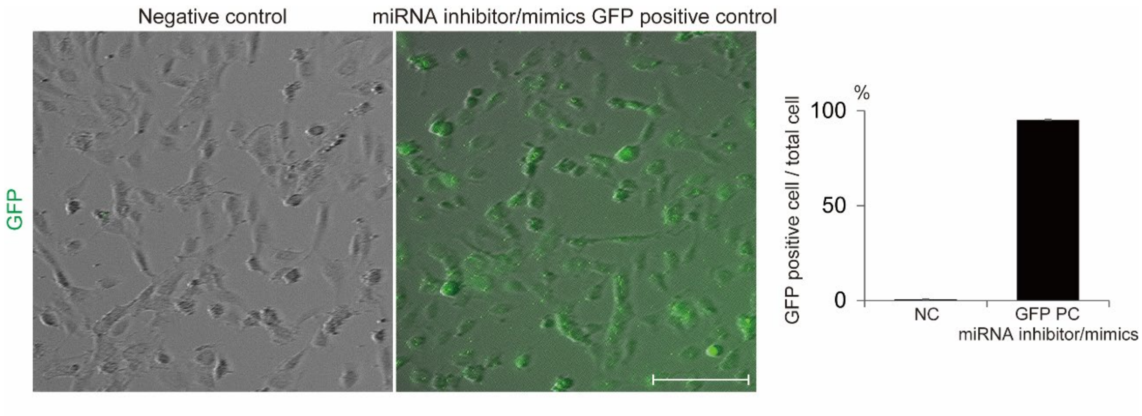

Figure S6. Tube formation assay using miRNA mimics, related to Figure 5.

miRNA mimics were added to the culture media for 24 hours. The number of tubes formed was then counted (n=3).

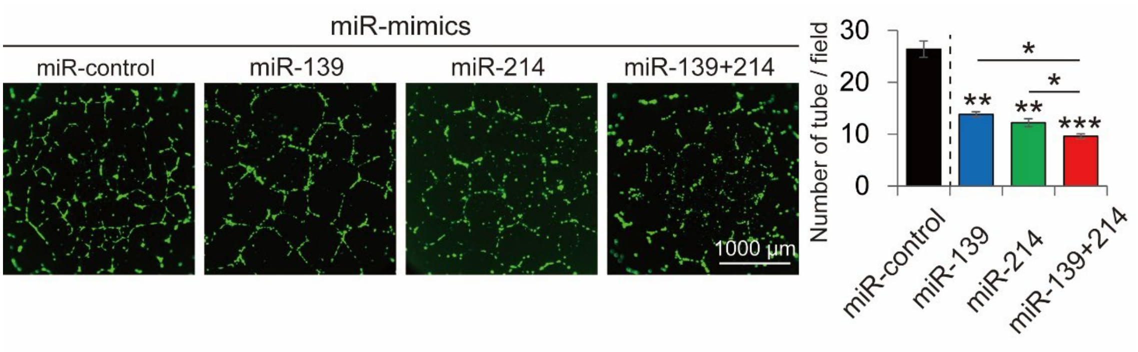

Figure S7. The pilot experiment of in vivo microRNA negative (NC) and positive control (PC) for the HCC orthotopic mouse model, related to Figure 6.

A) The expression of miR-PC (miR-1) was significantly increased in the tumor.

B) The expression of miR-PC has significantly increased in tumor endothelial cells (TEC)

C) The expression of Twf1, the gene targeting by miR-1, was significantly decreased in the tumor.

D) The expression of Twf1 was significantly decreased in TEC.

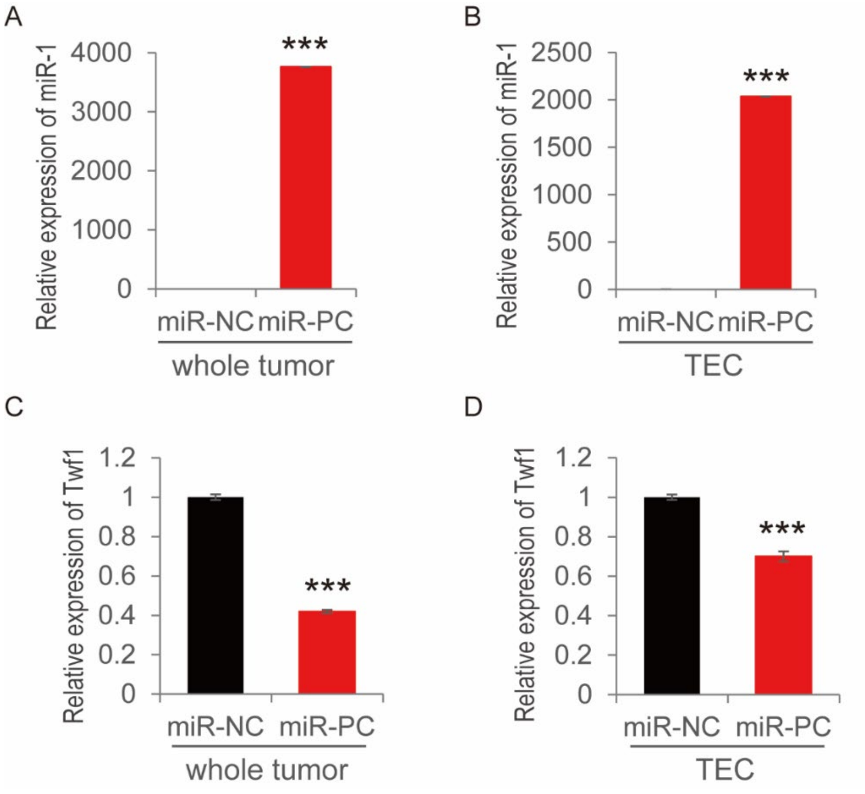

Supplement: Document S1. Figures S1–S7 and Tables S1 and S2 [file mmc1.pdf]
